# Correlation of thyroid-related hormones with vascular complications in type 2 diabetes patients with euthyroid

**DOI:** 10.3389/fendo.2022.1037969

**Published:** 2022-11-18

**Authors:** Jie Lin, Xin Xiang, Yahui Qin, Jing Gui, Qin Wan

**Affiliations:** ^1^ Department of Endocrinology and Metabolism, Affiliated Hospital of Southwest Medical University, Luzhou, China; ^2^ Metabolic Vascular Disease Key Laboratory of Sichuan Province, Luzhou, China; ^3^ Sichuan Clinical Research Center for Nephropathy, Luzhou, China; ^4^ Cardiovascular and Metabolic Diseases Key Laboratory of Luzhou, Luzhou, China

**Keywords:** free triiodothyronine, free thyroxine, thyroid-stimulating hormone, type 2 diabetes mellitus, diabetic peripheral neuropathy, carotid atherosclerosis

## Abstract

**Background:**

This study aimed to evaluate the relationship between thyroid-related hormones and vascular complications in type 2 diabetes mellitus (T2DM) patients with euthyroidism.

**Methods:**

We enrolled 849 patients with T2DM after screening out the ineligible. Multivariate logistic regression was used to analyze the relationship between fT3, fT4, the fT3/fT4 ratio, thyroid-stimulating hormone, and diabetic vascular complications. Spearman correlation analysis was used to determine the correlation between thyroid-related hormones and vascular complications.

**Results:**

In this cross-sectional study of T2DM, 538 patients with carotid atherosclerosis (CA) and 299 patients with diabetic peripheral neuropathy (DPN). The prevalence of DPN was negatively correlated with fT3 and the fT3/fT4 ratio but positively correlated with fT4 (all P<0.01). At the same time, the odds ratio for DPN decreased with increasing fT3 (T1: reference; T2: OR: 0.689, 95%CI: 0.477, 0.993; T3: OR: 0.426, 95% CI: 0.286, 0.633, all P<0.05) and fT3/fT4 ratio (T1: reference; T2: OR: 0.528, 95% CI: 0.365, 0.763; T3: OR: 0.413, 95% CI: 0.278, 0.613, all P<0.001). In terms of sensitivity and specificity, fT4 was found to be 39.5% and 71.4% accurate, respectively, with a 95% CI of 0.531-0.611.

**Conclusions:**

We found a negative correlation between fT3 and fT3/fT4 ratio and the number of individuals with DPN, and a positive correlation between fT4 and the prevalence of DPN.

## Introduction

One of the most frequent endocrine illnesses is diabetes mellitus (1). According to a recent report from the International Diabetes Federation (version 2019), the global prevalence of diabetes is expected to increase from 9.3% in 2019 to 10.9% in 2045 (2). More than 90% of people with diabetes have type 2 diabetes mellitus (T2DM) (3–5). The worldwide pandemic of type 2 diabetes indicates that more people are suffering from the psychological and physical burden of diabetes (6–8). T2DM can cause many vascular complications, such as coronary heart disease, renal disease, retinopathy, and diabetic peripheral neuropathy (DPN) (9, 10). Cardiovascular problems are the primary cause of death in T2DM patients (4). Therefore, other modifiable risk factors need to be investigation to prevent cardiovascular complications.

Like T2DM, thyroid diseases are also common endocrine system disorders (11). A growing number of studies suggest that abnormal thyroid function may impact glucose metabolism, insulin sensitivity, and the development of chronic complications of diabetes (12, 13). This indicates that T2DM and its long-term consequences may be associated with abnormal thyroid function (14, 15). Recent studies have shown that free triiodothyronine (fT3), free thyroxine (fT4) and thyroid-stimulating hormone (TSH) levels have been linked to insulin resistance and chronic complications of diabetes such as carotid atherosclerosis (CA) and DPN (16–19). However, patients with T2DM who have normal thyroid function have rarely been found to have a link between thyroid-related hormones and vascular problems like DPN and CA.

Therefore, the current study aimed to evaluate the association between fT3, fT4, fT3/fT4, TSH and DPN, CA in T2DM patients with euthyroidism.

## Methods

### Study subjects

A retrospective cross-sectional study was performed by the Department of Endocrinology and Metabolism at the Affiliated Hospital of Southwest Medical University on T2DM patients hospitalized in the department from 2018 to 2020. Inclusion criteria: (1) age > 18 years old; (2) T2DM diagnosis according to the American Diabetes Association “Standards of Medical Care in Diabetes” diagnostic criteria (2019 version (20).; (3) Normal thyroid function was defined as: fT3: 1.80–3.80 pg/mL, fT4: 0.78–1.86 ng/dL, TSH: 0.38–5.57 mIU/L. Exclusion criteria: (1) abnormal thyroid function test index; (2) a history of thyroid disease or thyroid surgery; (3) use of thyroxine preparations, amiodarone, and other drugs affecting thyroid function. Information for the study was collected by health professionals using a traditional questionnaire and validated equipment. Each participant provided demographic information and medical record information. We divided smokers into current smokers and nonsmokers. Similarly, alcohol drinkers were divided into current drinkers and non-drinkers. Mean arterial blood pressure was derived from three right arm arterial blood pressure measurements taken continuously for at least 30 min during a physical examination.

The study complied with the ethical standards of the Declaration of Helsinki (2013) and was approved by the Ethics Committee of the Affiliated Hospital of Southwest Medical University (ethical approval code: 2018017) (21). All enrolled participants signed the informed consent form.

### FT3/fT4 ratio assessment

FT3/fT4 ratio was determined as follows: fT3 (pg/mL)/fT4 (ng/dL). For further analysis, the fT3/fT4 ratio was transformed into categorical variables: T1: <1.88, T2: 1.88–2.24, T3: >2.24.

### Diagnosis of diabetic vascular complications

All subjects were scanned bilaterally in the carotid arteries by the same color Doppler ultrasound diagnostic instrument under the guidance of an ultrasound operator. During data collection, measurements were performed by a single operator. The criteria for atherosclerotic plaque formation were local carotid intima-media thickness (CIMT) ≥1.5 mm or local CIMT greater than 50% of the peripheral area. The diagnosis of carotid atherosclerosis was defined as an increase in CIMT ≥1.0 mm and/or the presence of carotid plaque (22–25). At least one symptom and/or one nerve conduction study neuropathy and aberrant signs are needed to diagnose DPN, as defined by the Toronto International Consensus for DPN (26).

### Statistics analysis

Descriptive statistics were used to compare baseline clinical characteristics of patients according to sex. For comparison between groups, one-way analysis of variance (ANOVA) (normally distributed continuous variables), Kruskal-Wallis H test (non-normally distributed continuous variables), and χ^2^ test (categorical variables) were used. Logistic regression analysis models were used to detect variables affecting diabetic complications. The relationship between thyroid-related hormones and vascular complications was identified by Spearman correlation analysis. We examined the anticipated accuracy of thyroid-related hormones using receiver operating characteristic (ROC) curves. All hypotheses were tested bilaterally at an α level of 0.05. The weighting of each risk factor was according to the values of odds ratio (OR). GraphPad Prism (version 9.0) was used to generate forest plots. SPSS (version 26.0) was used for all data analyses.

## Results

In this study, 869 patients with T2DM participated. There were 468 men and 401 women in the group. The mean age was 55.23 ± 11.14 years in men and 59.30 ± 10.23 years in women. and an average of 88.83 ± 73.43 months of diabetes on record. There were 538 patients with CA and 299 patients with DPN. The detailed demographic information and biochemical indicators of the participants are shown in **Table 1**. Compared with women, men had higher values of height, weight, body mass index (BMI), waist circumference (WC), diastolic blood pressure (DBP), alanine aminotransferase (ALT), FT3, FT3/FT4, visceral fat area (VFA), current smoking rates, current alcohol consumption, and use of antihypertensive medication (all P<0.05). Conversely, women had higher values for mean age, systolic blood pressure (SBP), high-density lipoprotein cholesterol (HDL), TSH, subcutaneous fat area (SFA), use of hypoglycemic drugs, and duration of diabetes (all P<0.05).

**Table 1:** Clinical characteristics according to gender.

Variables	Men (n=468)	Women (n=401)	P
Age, years old	55.23 ± 11.14	59.30 ± 10.23	<0.001*
Height, cm	166.09 ± 5.97	153.50 ± 6.01	<0.001*
Weight, kg	69.72 ± 10.36	58.27 ± 10.26	<0.001*
BMI, kg/m^2^	25.24 ± 3.22	24.68 ± 3.77	<0.018*
WC, cm	89.55 ± 9.74	84.95 ± 10.81	<0.001*
SBP, mmHg	134.47 ± 20.00	140.94 ± 21.06	<0.001*
DBP, mmHg	81.03 ± 11.26	78.74 ± 11.13	0.003*
TC, mmol/L	4.87 ± 3.58	4.95 ± 1.35	0.655
TG, mmol/L	2.63 ± 2.75	2.34 ± 2.24	0.087
HDL, mmol/L	1.09 ± 0.32	1.27 ± 0.40	<0.001*
LDL, mmol/L	2.78 ± 1.08	2.90 ± 1.09	0.097
FBG, mmol/L	9.22 ± 3.03	9.07 ± 3.28	0.487
HbA1c, %	9.78 ± 2.52	9.47 ± 2.31	0.056
ALT, mmol/L	31.73 ± 31.17	25.68 ± 23.44	0.001*
AST, mmol/L	25.37 ± 32.48	22.76 ± 15.93	0.143
FT3, pg/mL	2.59 ± 0.35	2.43 ± 0.32	<0.001*
FT4, ng/dL	1.25 ± 0.21	1.23 ± 0.20	0.165
FT3/fT4	2.12 ± 0.41	2.02 ± 0.40	<0.001*
TSH, mU/L	2.04 ± 1.08	2.43 ± 1.17	<0.001*
VFA	92.04 ± 44.26	86.24 ± 41.77	0.049*
SFA	159.74 ± 52.66	168.19 ± 64.75	0.034*
Duration of diabetes, months	82.41 ± 68.18	96.32 ± 78.55	0.005*
Current smoking (No/Yes)	225/243	394/7	<0.001*
Current drinking (No/Yes)	202/266	381/20	<0.001*
Antihypertensive drugs (No/Yes)	379/89	284/117	<0.001*
Hypoglycemic drugs (No/Yes)	190/278	136/265	0.043*
Insulin (No/Yes)	302/166	275/126	0.208

The values were expressed as the mean ± SD, n. BMI, body mass index; WC, waist circumference; SBP, systolic blood pressure; DBP, diastolic blood pressure; TC, total cholesterol; TG, triacylglycerol; HDL, high-density lipoprotein cholesterol; LDL, low-density lipoprotein cholesterol; FBG, fasting blood glucose; HbA1c, hemoglobin A1c; ALT, alanine aminotransferase; AST, aspartate aminotransferase; fT3, free triiodothyronine1; fT4, free thyroxine; TSH, thyroid stimulating hormone; VFA, visceral fat area; SFA, subcutaneous fat area. *P<0.05.

**Table 2** demonstrates the distribution of CA and DPN in the fT3, fT4, fT3/fT4 ratio, and TSH tertiles. The prevalence of CA in the fT3/fT4 tertiles was 66.2%, 56.0%, and 63.5%, respectively. Moreover, the prevalence of DPN negatively correlated with fT3 (42.7%, 33.6%, 26.8%, P<0.05) and fT3/fT4 ratio (46.9%, 30.9%, 25.3%, P<0.001), but positively correlated with fT4 (29.4%, 32.3%, 42.0%). Importantly, the prevalence of CA and DPN in all tertiles of TSH were virtually identical.

**Table 2:** Prevalence of CA and DPN in different thyroid-related hormones tertiles.

Events	Carotid atherosclerosis	P-value	Diabetic peripheral neuropathy	P-value
FT3				
T1 (1.80-2.36)	191 (65.2%)	0.343	125 (42.7%)	<0.001*
T2 (2.37-2.63)	178 (61.0%)	0.289	98 (33.6%)	0.024*
T3 (2.64-3.78)	169 (59.5%)	0.159	76 (26.8%)	<0.001*
FT4				
T1 (0.78-1.15)	191 (62.4%)	0.836	90 (29.4%)	0.004*
T2 (1.16-1.32)	177 (62.8%)	0.931	91 (32.3%)	0.453
T3 (1.33-1.85)	170 (60.5%)	0.633	118 (42.0%)	0.002*
FT3/fT4				
T1 (<1.88)	192 (66.2%)	0.032*	136 (46.9%)	<0.001*
T2 (1.88-2.24)	163 (56.0%)	0.012*	90 (30.9%)	<0.001*
T3 (>2.24)	183 (63.5%)	0.502	73 (25.3%)	<0.001*
TSH				
T1 (0.40-1.55)	186 (64.1%)	0.304	109 (37.6%)	0.239
T2 (1.56-2.48)	184 (63.2%)	0.820	90 (30.9%)	0.091
T3 (2.49-5.52)	168 (58.3%)	0.152	100 (34.7%)	0.474

The values were expressed as n (%). *P<0.05.

Multivariate regression models were used to calculate the odds ratios for DPN (**Table 3**). A higher fT3/fT4 ratio was associated with a lower odds ratio for DPN after adjustment for age and sex in model 2 (T1: reference; T2: OR: 0.482, 95% CI: 0.342, 0.680; T3: OR: 0.369, 95% CI: 0.258, 0.528; all P<0.001). This trend remained unchanged after adjustment for the remaining confounders in model 3 (T1: reference; T2: OR: 0.518, 95% CI: 0.352, 0.760; T3: OR: 0.356, 95% CI: 0.234, 0.542; all P<0.001). FT3 and the fT3/fT4 ratio were negatively related to DPN risk, whereas fT4 had the opposite effect. Unfortunately, we did not find a correlation between TSH and DPN prevalence.

**Table 3:** Adjusted OR and 95% CI in tertiles of thyroid-related hormones in the DPN group.

Events	Model 1	P-value	Model 2	P-value	Model 3	P-value
FT3						
T1	1	<0.001*	1	<0.001*	1	<0.001*
T2	0.679 (0.485,0.950)	0.024*	0.641 (0.455,0.903)	0.011*	0.689 (0.477,0.993)	0.046*
T3	0.491 (0.346,0.697)	<0.001*	0.433 (0.301,0.623)	<0.001*	0.426 (0.286,0.633)	<0.001*
FT4						
T1	1	0.004*	1	0.007*	1	0.092
T2	1.143 (0.805,1.623)	0.453	1.134 (0.797,1.612)	0.484	1.068 (0.737,1.550)	0.727
T3	1.773 (1.235,2.445)	0.002*	1.689 (1.198,2.382)	0.003*	1.467 (1.014,2.124)	0.042*
FT3/fT4						
T1	1	<0.001*	1	<0.001*	1	<0.001*
T2	0.507 (0.361,0.712)	<0.001*	0.482 (0.342,0.680)	<0.001*	0.528 (0.365,0.763)	0.001*
T3	0.384 (0.270,0.547)	<0.001*	0.369 (0.258,0.528)	<0.001*	0.413 (0.278,0.613)	<0.001*
TSH						
T1	1	0.239	1	0.293	1	0.443
T2	0.744 (0.527,1.049)	0.091	0.769 (0.543,1.087)	0.137	0.798 (0.554,1.150)	0.226
T3	0.883 (0.629,1.240)	0.474	0.949 (0.670,1.343)	0.766	0.954 (0.657,1.386)	0.807

Model 1: unadjusted; Model 2: adjusted for sex, age; Model 3: adjusted for sex, age, FBG, HbA1c, BMI, duration of diabetes, current smoking, current drinking, antihypertensive drugs, hypoglycemic drugs, insulin. Abbreviations: OR, odds ratio; CI, confidence interval. *P<0.05.

As shown in **Table 4**, the association between thyroid-related hormones and vascular complications in T2DM patients with euthyroidism was determined by Spearman correlation analysis. The results revealed a negative correlation between fT3 (rs=-0.144, P<0.001) and fT3/fT4 (rs=-0.193, P<0.001) and DPN, and a positive correlation between fT4 (rs=0.117, P=0.001) and DPN. However, we did not find any relationship between thyroid-related hormones and CA, and the relationship between TSH and DPN.

**Table 4:** Association among thyroid-stimulating hormone and vascular complications.

Events	CA, rs	P	DPN, rs	P
FT3	-0.061	0.073	-0.144	<0.001*
FT4	-0.018	0.594	0.117	0.001*
FT3/fT4	-0.021	0.541	-0.193	<0.001*
TSH	-0.064	0.060	-0.025	0.455

fT3, free triiodothyronine1; fT4, free thyroxine; TSH, thyroid stimulating hormone; CA, carotid atherosclerosis; DPN, diabetic peripheral neuropathy; rs, Spearman’s correlation coefficient. *P<0.05.

**Figure 1:**
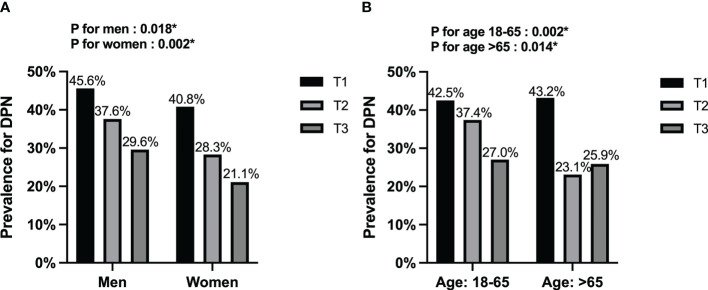
Stratification analysis of DPN among the fT3 tertiles. **(A)** Stratified by sex; **(B)** Stratified by age. DPN, diabetic peripheral neuropathy. *P<0.05.

Furthermore, we used fT3 tertiles to separate the DPN groups by, sex and age (**Figure 1**). The results showed that the overall prevalence was higher in men than in women. Meanwhile, all DPN subgroups, except those aged > 65 years, showed a declining prevalence with increasing fT3 (P<0.05).

**Figure 2:**
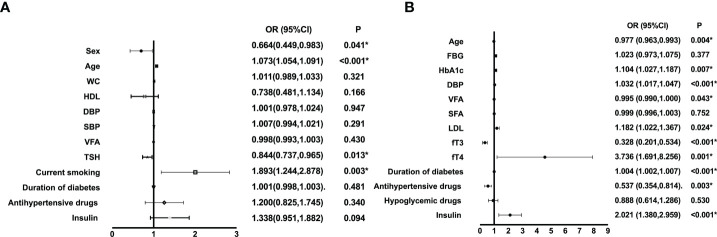
Multiple regression analysis of variables independently associated with CA **(A)** or DPN **(B)** in all participants. WC, waist circumference; SBP, systolic blood pressure; HDL, high-density lipoprotein cholesterol; FBG, fasting blood glucose; HbA1c, hemoglobin A1c; DBP, diastolic blood pressure; VFA, visceral fat area; SFA, subcutaneous fat area; LDL, low-density lipoprotein cholesterol; fT3, free triiodothyronine1; fT4, free thyroxine; TSH, thyroid stimulating hormone; CA, carotid atherosclerosis; DPN, diabetic peripheral neuropathy. *P<0.05.

As illustrated in **Figure 2**, we performed multivariate regression analysis for variables independently associated with diabetic CA or DPN. CA risk factors were age (OR: 1.073, 95% CI: 1.054, 1.091) and current smoking (OR: 1.893, 95% CI: 1.244, 2.878) (all P<0.01). And the forest plot revealed that HbA1c (OR: 1.104, 95% CI: 1.027, 1.187), DBP (OR: 1.032, 95%CI: 1.017,1.047), low-density lipoprotein cholesterol (LDL) (OR: 1.182, 95% CI: 1.022, 1.367), fT4 (OR: 3.736, 95%CI: 1.691,8.256), duration of diabetes (OR: 1.004, 95% CI: 1.002, 1.007), and insulin use (OR: 2.021, 95% CI: 1.380, 2.959) were risk factors for DPN (all P <0.05). Moreover, age (OR: 0.977, 95% CI: 0.963, 0.993) and antihypertensive drugs use (OR: 0.537, 95% CI: 0.354, 0.814) were protected factors for DPN (all P <0.01).

**Figure 3:**
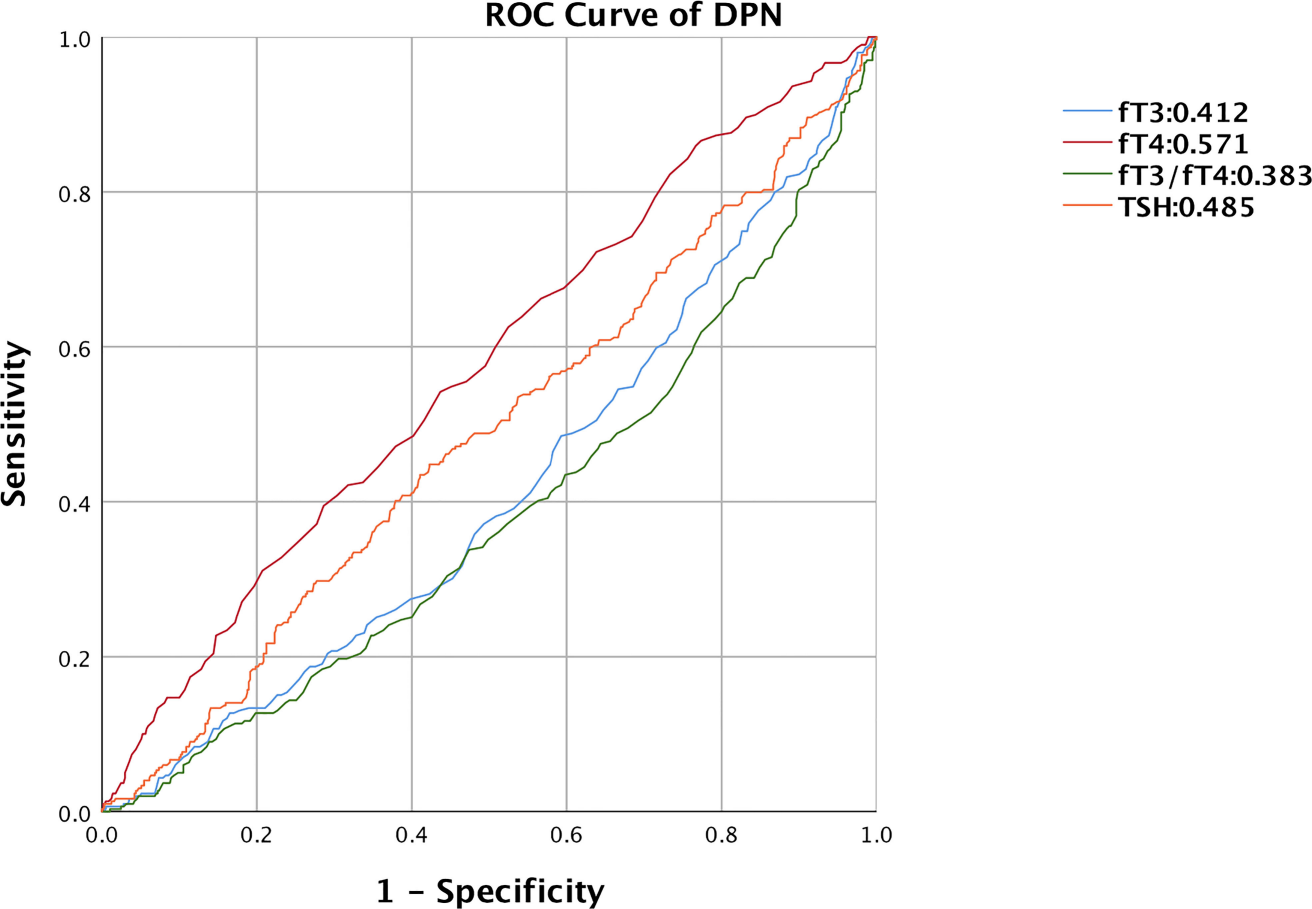
ROC curve of thyroid-related hormones predicting DPN in T2DM with euthyroid. ROC, receiver operating characteristic; DPN, diabetic peripheral neuropathy; fT3, free triiodothyronine1; fT4, free thyroxine; TSH, thyroid stimulating hormone.

Finally, we assessed the diagnostic value of thyroid-related hormones for DPN using a ROC curve (**Figure 3**). With an under the curve (AUC) of 0.571 (95% CI: 0.531, 0.611, P<0.001), fT4 was found to be the most accurate, followed by TSH (AUC: 0.485, 95%CI: 0.444, 0.525, P=0.455), fT3 (AUC: 0.412, 95%CI: 0.373, 0.452, P<0.01), fT3/fT4 ratio (AUC: 0.383, 95%CI: 0.343, 0.422, P<0.001). By calculating the Jorden index, the optimum cutoff value of fT4 was 1.325. The sensitivity of fT4 was 39.5%, and the specificity was 71.4%.

## Discussion

This cross-sectional study included 869 T2DM patients. We explored the correlation between the prevalence of DPN and CA and fT3, fT4, fT3/fT4, and TSH in T2DM patients with normal thyroid function. Significantly fewer people in the higher fT3 or fT3/fT4 tertiles had DPN (P<0.001). However, the prevalence of DPN increased with increasing fT4 tertiles (P<0.01). After adjustment for confounding factors, fT3 and the fT3/fT4 ratio was negatively associated with DPN (P < 0.05). Overall, fT3 performed the best in assessing the correlation of thyroid hormones with the prevalence of DPN, followed by fT3/fT4 ratio and fT4. Interestingly, we found DPN is more associated with thyroid hormone (TH) levels than with TSH levels.

DPN is a common microvascular complication of diabetes leading to lower limb amputations, neuropathic pain, and increased mortality (27). Both T2DM and obesity exacerbate the inflammatory response, leading to increased serum concentrations of inflammatory biomarkers such as C-reactive protein (28–30). These inflammatory biomarkers diminish the autonomic function and promote the development and progression of neuropathy (31). This may explain why LDL-c is a risk factor for DPN. Yang et al. demonstrated a linear relationship between T2DM and patients with HbA1c > 7.0% and an increased risk of DPN (32). This is because hyperglycemia and insulin resistance are essential for the development of DPN (27, 33, 34). In this study, DBP was found to be independently associated with the risk of developing DPN. The exact mechanism is unclear, and possibly due to a combination of reduced neural blood flow, slow nerve conduction, axonal atrophy, and thinning of myelinated fibers as a result of elevated blood pressure (35–37). The use of antihypertensive drugs may reverse these effects, leading to the annihilation of the risk of DPN. Several studies have shown that the prevalence of DPN increases with the duration of disease in T2DM, suggesting that we should screen for DPN early in the T2DM population (38–40). Patients with poor glycemic control may develop insulin neuritis characterized by acute severe distal limb pain, peripheral nerve fiber damage, and autonomic dysfunction (41, 42). Although rare, these patients are more likely to develop pain or autonomic neuropathy (42).

CA manifests as plaque formation and stenosis of the carotid artery and is a common macrovascular complication of T2DM, the prevention and treatment of which are of critical importance because of its high lethality (43). Some studies have demonstrated that smoking promotes CA by increasing the intima-medial wall thickness of the carotid artery (44). In addition, diabetics are at high risk for arterial atherosclerosis (45) because the arterial walls become less pliable and even harden with age. Plaque composed of cholesterol, fat, calcium, and fibrous tissue accumulates, causing blood vessels to narrow and promoting atherosclerosis (46, 47). Zhou et al. identified a negative correlation between fT3 and carotid intima-media thickness (17). Our study provides evidence to confirm their findings.

The mechanisms concerning fT3 and DPN pathogenesis are unclear. Some investigators have suggested that degeneration of microvascular endothelial cells and peripheral endothelial cells is one of the characteristic changes of DPN (48–50). In addition, the decrease in NO, which can relax blood vessels, is associated with endothelial dysfunction (51, 52). Some studies have reported that T3 mediates the production of endothelial NO (53). Therefore, T3 may reduce the occurrence of DPN by protecting the endothelial cells of microvessels. In addition, the natural metabolite 3,5-diiodothyronine, generated by the deiodination pathway of T3, could reverse the process of DPN by regulating the expression of Sirtuin 1 protein (54, 55). Previous studies have shown that fT3 level was negatively associated with risk of abnormal nerve conduction (56). Meanwhile, Li et al. demonstrated a negative association of fT3 with DPN in T2DM patients with normal thyroid function (57). This is consistent with our findings.

FT4 is deiodinated by three deiodinases to form the more active fT3, which exerts potent biological effects on target organs and tissues (58). Type II deiodinase (DIO2) is the most potent in converting T3, and fT3/fT4 is an indicator of DIO2 activity (59). Intracellular activation of thyroid hormone *via* DIO2 attenuates cellular dependence on aerobic glycolysis, decreases ROS production and thus minimizes oxidative stress (60). Therefore, decreased DIO2 activity and fT3 conversion lead to increased oxidative stress, which in turn promotes an inflammatory response. Further, prolonged exposure to a hyperglycemic environment promotes oxidative stress (61). This suggests that T2DM patients have decreased DIO2 activity, increased fT4, decreased fT3 production, and enhanced oxidative stress and inflammatory responses, which promote the development of DPN (61–63). Foremost, fT4 level was an independent risk factor for DPN (OR:3.376, 95% CI: 1.691,8.256) in our study. Hu et al. showed that fT4 was negatively correlated with DPN (OR:0.800) (64). This is contrary to our findings. The possible reasons include their smaller sample size, only containing 248 patients with T2DM, and group variability, living environment may have played a role.

When subclinical hypothyroidism occurs, it can exacerbate abnormalities in glucolipid metabolism and significantly exacerbate DPN due to TSH receptor palmitoylation-induced oxidative stress and apoptosis in Schwann’s cells (65). A cross-sectional study from China also demonstrated that TSH levels were independently associated with DPN in the T2DM population in patients with subclinical hypothyroidism (19). However, our study found that this correlation disappeared when the patient’s thyroid function was at normal levels. Does this mean that we should control TSH levels in patients with DPN? In addition, TSH can exacerbate atherosclerosis by promoting macrophage inflammation in plaques (66). Several studies have found a positive correlation between TSH and carotid intima-media thickness in patients with normal thyroid function (67, 68). Interestingly, we did not find this correlation in patients with T2DM. As we all know, hyperglycemia, obesity, and dyslipidemia can promote inflammation and exacerbate CA. The exact mechanism is unclear, and more animal studies are needed to explore it.

As we all know, early detection, early diagnosis, and early treatment can stop and delay the development of the disease and help patients return to health as soon as possible. If the high-risk group of DPN can be detected in advance to help patients diagnose as well as treat them earlier, the development of organic diseases such as gangrene can be reduced or avoided. For people with T2DM, regular monitoring of glycosylated hemoglobin to regulate blood glucose is essential. We can have the treating physician screen the patient for glycosylated hemoglobin along with thyroid function. Reducing the incidence of DPN by controlling fT4 at low normal levels or fT3 at high normal levels after thyroid disease has been ruled out as the cause of the abnormal thyroid function.

In this study, several thyroid-related hormones (fT3, fT4, fT3/fT4, TSH) were evaluated for their association with DPN or CA. Previous research often examined only one of these hormones in relation to the thyroid. One limitation of our study was its a small sample size, which could have led to some errors in the results. Furthermore, it was a single-center cross-sectional study, so the findings cannot be directly attributed to a specific cause. Therefore, further multicenter cohort studies are urgently required to support our conclusions.

## Conclusions

Individuals with a low fT3 level and fT3/fT4 ratio in T2DM with normal thyroid function and those with high fT4 levels were more likely to develop DPN. Considering that elevated fT4 is a risk factor for DPN, even in the euthyroid range, early control of fT4 levels or redefinition of its reference range (0.78-1.15 ng/dL) in the T2DM population is beneficial for the prevention of DPN. And fT3 should be early controlled at high normal levels (2.64-3.78 pg/mL).

## Data availability statement

The original contributions presented in the study are included in the article/supplementary material. Further inquiries can be directed to the corresponding author/s.

## Ethics statement

The studies involving human participants were reviewed and approved by the ethics committee of the Affiliated Hospital of Southwest Medical University (ethical approval code: 2018017). The patients/participants provided their written informed consent to participate in this study.

## Author contributions

JL, XX, and YQ made equal contributions to this work, performing the statistical analysis, interpreting the results, and writing the paper. JG reviewed the data, and QW proposed the ideas. All authors contributed to the article and approved the submitted version. 

## Funding

The Ministry of Science and Technology of China provided funding for this study through grants 2016YFC0901200.

## Acknowledgments

Thanks to Yao, Liu, Linlin, Hang and Hongya, Wang for their help in clinical data analysis.

## Conflict of interest

The authors declare that the research was conducted in the absence of any commercial or financial relationships that could be construed as a potential conflict of interest.

## Publisher’s note

All claims expressed in this article are solely those of the authors and do not necessarily represent those of their affiliated organizations, or those of the publisher, the editors and the reviewers. Any product that may be evaluated in this article, or claim that may be made by its manufacturer, is not guaranteed or endorsed by the publisher.
